# BEMER Therapy Combined with Physiotherapy in Patients with Musculoskeletal Diseases: A Randomised, Controlled Double Blind Follow-Up Pilot Study

**DOI:** 10.1155/2015/245742

**Published:** 2015-05-20

**Authors:** Franciska Gyulai, Katalin Rába, Ildikó Baranyai, Enikő Berkes, Tamás Bender

**Affiliations:** Hospitaller Brothers of St. John of God, Árpád fejedelem útja 7, Budapest 1023, Hungary

## Abstract

*Background.* This study evaluates the effect of adjuvant BEMER therapy in patients with knee arthrosis and chronic low back pain in a randomized double blind design. *Methods*. A total of 50 patients with chronic low back pain and 50 patients with osteoarthritis of knee took part in this study and were randomized into 4 groups. Hospitalized patients received a standardized physiotherapy package for 3 weeks followed by BEMER therapy or placebo. *Results*. In patients with low back pain, the comparison of the results obtained at the first and second visit showed a significant improvement in resting VAS scores and Fatigue Scale scores. The Oswestry scores and Quality of Life Scale scores showed no change. In patients with knee arthrosis, the comparison of the first and second measurements showed no significant improvement in the abovementioned parameters, while the comparison of the first and third scores revealed a significant improvement in the Fatigue Scale scores and in the vitality test on the Quality of Life Scale. *Conclusions*. Our study showed that BEMER physical vascular therapy reduced pain and fatigue in the short term in patients with chronic low back pain, while long-term therapy appears to be beneficial in patients with osteoarthritis of knee.

## 1. Introduction 

Electromagnetic field has been used in healing for centuries and has a medical literature of many decades, as well [1]. During the 1960s, Bassett confirmed that this therapy has a stimulating effect on callus formation and thus one aim of the study was to evaluate the effect of pulsed electromagnetic field on osteoblastic activity both* in vitro* and* in vivo* [2]. There are only a few areas of physiotherapy that are so controversial in the medical community as this therapy. Many people refer to it only as an alternative therapy, while others see it as a treatment for a number of conditions. One reason for this is that prominent medical journals publish articles expressing completely opposed positions on the effects of magnetic therapy used in a specific indication. (Pulsed electromagnetic field generators use different signal formats, so they produce different effects. Identical impulse format is for this therapy what identical active substance is for medicines.) There are many data available for both ultrasound and TENS as conventional physical therapies; however, these evidences are not convincing [3, 4]. As regards electromagnetic therapies, pulsed magnetic therapy is widely used, unlike therapy in static magnetic fields. In the case of pulsed electromagnetic field (PEMF), a number of different frequency ranges can be used. One of the assumed mechanisms of action of the electromagnetic field is the ion cyclotron resonance effect, through the modulation of ion bindings, an effect on free radicals, and an effect on heat shock proteins. The beneficial effect on angiogenesis may play a role in the facilitation of callus formation [5]. PST (Pulsed Signal Therapy) is different from PEMF as PST is an extended version of PEMF, whose beneficial effects on human chondrocytes were confirmed by* in vitro* studies [6]. Moreover, PEMF also has a chondroprotective effect [7]. BEMER (Bio-Electro-Magnetic-Energy-Regulation) devices operate with special parameters, and the “weak” magnetic field is only a vehicle and a special pulsed signal was developed to this end (BEMER signal), the primary effect of which is an improvement in tissue microcirculation. In contrast to the known magnetic field wave patterns that can easily be described by mathematical formulae, the BEMER therapy developed by J. Klopp essentially applies the specifically developed BEMER signal patterns. As a result, a significant increase in the vasomotion of microvessels, arteriovenous pO2 difference, number of open capillaries, arteriolar and venular flow volume, and flow rate of red blood cells is observed in a specific microcirculatory area. This change in the microcirculation status was demonstrated by combining high-resolution intravital microscopy, computer image processing, and measurement of microflow rate using laser reflection spectroscopy [8, 9]. BEMER devices generate a maximum magnetic induction of 100–150 *μ*T; for comparison, the magnetic field of Earth in Budapest is approximately 47-48 *μ*T.

Treatment time is usually 20 minutes a day (depending on the applicator) for 3-4 weeks depending on the diagnosis. Improvement of microcirculation and reducing fatigue are the clinical applications that have so far been confirmed.

*Aim of the study was* to evaluate the effect of adjuvant BEMER therapy on pain, fatigue, and quality of life in patients with knee arthrosis and chronic low back pain. The* primary outcomes* were to assess the effect of BEMER therapy on knee and low back pain caused by degenerative changes. The* secondary outcomes* were to evaluate the adverse effects and to record the changes in fatigue and to investigate the effect on quality of life.

## 2. Materials and Methods

### 2.1. Study Design

This is a single-centre, randomized, placebo-controlled, double blind follow-up study. A total of 50 patients with chronic low back pain and 50 patients with knee arthrosis were enrolled in this study who had been hospitalized for 3 weeks at the Rheumatologic Rehabilitation Department of the Hospitaller Brothers of St. John of God.

#### 2.1.1. Ethics

The patients signed an Informed Consent Form before the study. The study was approved by the Ethics Committee.

#### 2.1.2. Procedure

In addition to complex standard physiotherapy, half of the patients also received additional BEMER therapy, while the other half received additional placebo BEMER therapy; patients could not tell the placebo treatment from the real treatment. Randomisation was conducted by an independent person (by means of drawing lots). During this study, neither the study doctor nor patients or study assistants knew the treatment given. Unblinding took place only after study completion.

#### 2.1.3. Participations

Demographics: average age was 67.29 years ± 5.44 years (males) and 66.7 years ± 7.73 years (females) in patients with chronic low back pain and 67.11 years ± 8.8 years (males) and 65.3 years ± 7.46 years (females) in patients with osteoarthritis of knee; with 2 and 3 exceptions, all patients with chronic lower back pain and with osteoarthritis of knee, respectively, were females. There were no differences in gender and age between the treatment and placebo group.

*Inclusion Criteria for Patients with Low Back Pain*. These include

- patients with chronic nonspecific low back pain with nonseverely reduced mobility;
- males and females of 20 to 80 years of age;
- nonspecific low back pain for at least 12 weeks;
- palpable tenderness of the paravertebral muscles and/or painful limited mobility of the lumbar spine;
- low back pain VAS (visual analogue scale) score of at least 30 mm on a 100 mm visual analogue scale during exercise;
- the patients have not received systemic or local steroid therapy or physical therapy or balneotherapy, within 2 months prior to the study; physiotherapy was allowed.

*Exclusion Criteria for Patients with Low Back Pain*. These include

- acute low back pain;
- organic neurological deficit associated with lower back pain;
- the underlying cause is likely to be vertebral compression fracture caused by osteoporosis or other factors;
- underlying malignancy;
- pain caused by inflammatory spine conditions;
- spondylolisthesis (grade 2 or higher);
- pregnancy.

*Inclusion Criteria for Patients with Osteoarthritis of Knee*. These include

- males and females of 30 to 80 years of age with mild or moderate knee arthrosis reporting knee pain characteristic of arthrosis for at least 3 months;
- diagnosis of knee arthrosis confirmed by imaging meeting ACR (American College of Rheumatology) criteria [10].

*Exclusion Criteria for Patients with Osteoarthritis of Knee*. These include

- inflammatory rheumatic conditions;
- palpable effusion in the knee;
- knee injury within 6 months prior to the study;
- intra-articular steroid within 1 month prior to the study;
- intra-articular hyaluronic acid within 6 months prior to the study;
- patients with femoral neuralgia or radiculopathy;
- NSAID (nonsteroidal anti-inflammatory drug) therapy or chondroprotective therapy modified within 1 month prior to the treatment;
- knee surgery within 6 months;
- pregnancy.

*Intervention.* All patients were administered a standardized physiotherapy package during the study (individual and group exercises (30 minutes): underwater whirlpool massage (10 minutes), TENS therapy on the low back or knee (15 minutes every day), and aquagym (30 minutes every other day)).

In addition to the standard complex physical therapy 50% of the patients received BEMER therapy, whereas 50% received placebo BEMER therapy.

Each BEMER session lasted 20 minutes; parameters were using mattress applicator (B. Body Pro): 7–35 microTesla, intensive applicator (B. PAD): 60–100 microTesla, or mattress applicator (B. Body Pro) intensity levels 2-3-4–10, intensive applicator (B. PAD) intensity levels 6-7-8-9-10 and using vascular motion signal configuration (BEMER signal). The device was a BEMER International AG (Liechtenstein) product. Accessories were mattress with therapy unit (B. Body), flexible, intensive, small surface unit (B. PAD) and B. SPOT (intensive point-like unit), and B. LIGHT unit as needed (may be connected to light therapy unit). The B. BOX Professional control units have 10 different levels of intensity and 3 predefined programmes. The intensity levels are applied during the general full body surface treatment according to the basic programme, while programmes P1–P3 are used gradually to achieve the “deep effect” during targeted treatments. Patients in both groups were treated in supine position while receiving B. BODY mattress applicator treatment. Low back pain patients received B. PAD therapy placed in the low back region at the same time as the full body treatment. Knee pain patients had the B. PAD applicator placed on their knees at the same time as the full body treatment. Therapy sessions lasted 20 minutes each with the B. BODY and B. PAD applicator, respectively:

 parameters evaluated: pain intensity on a visual analogue scale (VAS) of 10 cm;
 General Quality of Life Questionnaire SF 36 [11, 12];
 Facit Fatigue Scale (fatigue intensity ranged from 1 to 50) [13];
 Oswestry Index for patients with low back pain [14, 15];
 WOMAC Index for patients with knee pain [16, 17].

We used the data from only those patients who received at least 12 sessions of treatment (each patient completed 15 sessions).

*Study Procedure*. It is as follows.

1. Before starting the therapy, the physician takes a detailed history, performs a physical examination, checks whether the patient meets the inclusion and exclusion criteria, informs the patient about the study, obtains a signed Informed Consent Form, and administers the questionnaires (WOMAC Index or Oswestry Index and SF 36, and VAS and Fatigue Scale).
2. At the end of the therapy sessions, the physician examines the patient, administers the abovementioned questionnaires, and asks about the adverse effects.
3. Follow-up period after 15 weeks (the patient has returned the self-administered WOMAC, Oswestry, and SF36 questionnaires).

### 2.2. Statistical Analysis

The analyses focused on pairwise comparisons among the two selected study arms based on the intention-to-treat (ITT) principle. The One-Sample Kolmogorov-Smirnov Test was applied for testing normality. Analysis of covariance (ANCOVA) was used with the analgesic as a covariate to measure effectiveness by comparing study arms. The significance level was set at alpha = 0.05 (two-tailed). All analyses were carried out using the R-software version 2.9.1 (R Development Core Team, 2009).

## 3. Results

### 3.1. Low Back Pain

Of the 25 patients with low back pain, 4 patients (2 cases with acute fever and 2 older patients have misunderstood how to fill out the questionnaires) and 6 patients (2 cases with acute fever and 4 patients had poor compliance) were excluded from the placebo group and the treatment group, respectively, after the second measurement. Of the patients with knee arthrosis, 2 patients (because of gastroenteritis) and 6 patients (2 cases with acute fever and 4 patients whose questionnaires could not be evaluated) were excluded from the placebo group and the treatment group, respectively. No adverse effects were seen. In the group with low back pain, the comparison of the results from the first and second visit showed a significant improvement in resting VAS scores and Fatigue Scale scores and the exercise VAS scores were close to the level of significance, while no changes were found in the Oswestry scores and Quality of Life (see Table 1). Based on the comparison between the first and third measurements, there was no significant change in either value (see Table 2).

### 3.2. Knee Osteoarthritis

As regards knee complaints, the comparison of the results of the first and second measurements showed no significant improvement in either parameter (moreover, VAS scores were better in the placebo arm) (see Table 3). Based on the comparison of the first and third measurements, scores on the Fatigue Scale improved significantly, just as the vitality score on the Quality of Life scale (see Table 4). There were no changes in medication.

## 4. Discussions

Preliminary data suggest that BEMER therapy may have a pain-relieving and fatigue-reducing effect in the treatment of chronic low back pain, even in the short term (studies conducted on large patient populations are required to confirm this). However, for long-term improvement, the therapy should be applied for a long period of time (but to prove this, also further examinations are required.). There was no short-term beneficial effect during knee therapy, probably due to the fact that Program P2 should be used instead of Program P3 (as the deep effect is not that pronounced, however, this is only a hypothesis and needs further studies to be demonstrated), although it was effective in the long term. In this study, we used BEMER therapy not as monotherapy but as adjuvant physiotherapy for inpatients. Many studies with pulse electromagnetic field (PEMF) in patients with locomotor diseases have been published. In patients with osteoarthritis of knee, PEMF was administered as adjuvant therapy in a total of 483 patients in 9 studies, which showed an improvement in the total clinical score [18]. Based on 14 studies included in another review article, significant improvement in knee arthritis was seen after 8 weeks as compared to patients receiving placebo [19]. Turkish authors administered PEMF therapy in addition to ultrasound treatment and physiotherapy, but there was no difference between the two groups (those who received PEMF as adjuvant therapy and those who received no additional PEMF therapy) [20]. In patients with osteoarthritis of knee, a static magnetic knee protector with a field of 35 mT was used for 12 weeks (placebo-controlled study). There were no differences between the two groups in the outcome parameters [21]. In a double blind controlled study conducted in patients with fibromyalgia, although the number of cases was limited, a significant pain-relieving effect was confirmed following treatment with a weak electromagnetic field [22]. Hungarian authors studied thirty patients with obliterative vascular disease of the lower limb. They measured pain-free and maximum walking distance using a treadmill. After the placebo period, the patients were administered 8 sessions of BEMER physical vascular therapy, then i.v. pentoxiphylline therapy. Pain-free and maximum walking distance was measured after each session of therapy. As a result of BEMER physical vascular therapy, pain-free and maximum walking distance increased by 57.4%. Combined therapy (BEMER physical vascular therapy + rheological therapy) increased the measured values by 81.9% and 84.0%, respectively. Combined therapy led to a significant improvement in the walking distance as compared to the pretherapy level [23]. According to a double blind controlled study, BEMER therapy (2 × 8 minutes for 12 weeks) alleviated fatigue in patients with multiple sclerosis; subsequently, a 3-year open-label trial confirmed the long-term effect [24, 25]. A double blind study involving musculoskeletal patients was first published in 2009 [26].

### 4.1. Limitations of the Study

This is a pilot study. Unfortunately, many of the patients failed to return or misunderstood how to fill out the questionnaires during the three-month period for returning them, which led to a decrease in the number of cases. Furthermore, the Fatigue Scale includes many questions that are uncharacteristic of inpatients and can be evaluated only in outpatients: if, for example, a patient fails to answer Questions 2, 3, and 4, the entire questionnaire will be unevaluable (the software will ignore it). Some patients failed to completely fill out the SF 36 and the Fatigue Scale because they did not perform a specific physical activity during hospitalization. In the case of SF 36, patients failed to answer 1 or 2 questions in a number of item groups. They either did not understand the questions or were not allowed to perform a specific activity. Unfortunately, these cases also led to worse results because more significant improvements would have been possible in many cases in a larger study population.

## 5. Conclusions

Our study suggests the possibility that BEMER therapy administered in combination with traditional physiotherapy procedures reduces chronic lower back pain in the short term and may be effective in the long-term treatment of patients with osteoarthritis of knee. However, well-performed studies with a larger sample size are required for a more exact evaluation of the abovementioned effects.

## Figures and Tables

**Table 1:** Comparison of values before and after treatment of low back pain.

Dependent variable	Therapy	Descriptive statistics			*p* values of ANCOVA
		Mean	Std. deviation	*N*	
Resting VAS diff. Tests 1 and 2	Physio + BEMER	26.84	13.68	19	0.0229
	Physio + placebo	15.00	15.23	21	
Resting VAS rel. Tests 1 and 2	Physio + BEMER	0.55	0.28	18	0.0620
	Physio + placebo	0.35	0.44	18	

Exercise VAS val. diff. Tests 1 and 2	Physio + BEMER	29.79	15.11	19	0.0547
	Physio + placebo	21.33	17.06	21	
Exercise VAS val. rel. Tests 1 and 2	Physio + BEMER	0.44	0.23	18	0.0179
	Physio + placebo	0.30	0.28	20	

Oswestry diff. Tests 1 and 2	Physio + BEMER	9.80	8.16	19	0.6872
	Physio + placebo	9.27	11.72	21	
Oswestry rel. Tests 1 and 2	Physio + BEMER	0.24	0.18	18	0.8190
	Physio + placebo	0.21	0.28	20	

Fatigue GFI diff. Tests 1 and 2	Physio + BEMER	12.40	9.53	9	0.0218
	Physio + placebo	6.71	9.22	9	
Fatigue GFI rel. Tests 1 and 2	Physio + BEMER	0.32	0.28	8	0.0153
	Physio + placebo	0.22	0.23	7	

Physical functioning nbs diff. Tests 1 and 2	Physio + BEMER	−3.45	3.17	15	0.4952
	Physio + placebo	−3.15	6.95	14	
Physical functioning nbs rel. Tests 1 and 2	Physio + BEMER	−0.10	0.10	14	0.5188
	Physio + placebo	−0.09	0.22	13	

Role physical nbs diff. Tests 1 and 2	Physio + BEMER	−13.17	14.35	15	0.7722
	Physio + placebo	−8.42	14.84	16	
Role physical nbs rel. Tests 1 and 2	Physio + BEMER	−0.49	0.63	14	0.8037
	Physio + placebo	−0.37	0.59	15	

Bodily pain nbs diff. Tests 1 and 2	Physio + BEMER	−14.57	9.75	15	0.1431
	Physio + placebo	−7.68	8.05	19	
Bodily pain nbs rel. Tests 1 and 2	Physio + BEMER	−0.44	0.36	14	0.2485
	Physio + placebo	−0.24	0.25	18	

General Heath nbs diff. Tests 1 and 2	Physio + BEMER	−2.81	8.23	12	0.9361
	Physio + placebo	−2.74	6.06	17	
General Heath nbs rel. Tests 1 and 2	Physio + BEMER	−0.09	0.27	11	0.9707
	Physio + placebo	−0.10	0.20	16	

Vitality nbs diff. Tests 1 and 2	Physio + BEMER	−7.92	6.86	12	0.6338
	Physio + placebo	−5.01	5.05	16	
Vitality nbs rel. Tests 1 and 2	Physio + BEMER	−0.20	0.20	11	0.6240
	Physio + placebo	−0.12	0.13	15	

Social functioning nbs. diff. Tests 1 and 2	Physio + BEMER	−7.33	8.59	13	0.7860
	Physio + placebo	−4.75	9.37	19	
Social functioning nbs. rel. Tests 1 and 2	Physio + BEMER	−0.23	0.28	13	0.8866
	Physio + placebo	−0.17	0.34	18	

Role emotional nbs diff. Tests 1 and 2	Physio + BEMER	−12.93	17.67	14	0.3680
	Physio + placebo	−7.84	14.36	16	
Role emotional nbs rel. Tests 1 and 2	Physio + BEMER	−0.62	1.07	13	0.4572
	Physio + placebo	−0.41	0.89	15	

Mental health nbs diff. Tests 1 and 2	Physio + BEMER	−8.28	8.77	12	0.5300
	Physio + placebo	−6.05	8.85	16	
Mental health nbs rel. Tests 1 and 2	Physio + BEMER	−0.22	0.28	11	0.9709
	Physio + placebo	−0.17	0.31	15	

Physical component summary diff. Tests 1 and 2	Physio + BEMER	−4.99	4.45	7	0.1974
	Physio + placebo	−6.04	9.23	12	
Physical component summary rel. Tests 1 and 2	Physio + BEMER	−0.14	0.13	7	0.1514
	Physio + placebo	−0.22	0.34	11	

Mental component summary diff. Tests 1 and 2	Physio + BEMER	−7.82	11.09	7	0.6788
	Physio + placebo	−5.38	8.76	12	
Mental component summary rel. Tests 1 and 2	Physio + BEMER	−0.26	0.42	7	0.6899
	Physio + placebo	−0.16	0.27	11	

**Table 2:** Comparison of values before and 3 months after treatment of low back pain.

Dependent variable	Therapy	Descriptive statistics			*p* values of ANCOVA
		Mean	Std. deviation	*N*	
Resting VAS diff. Tests 1 and 3	Physio + BEMER	15.94	22.98	18	0.7766
	Physio + placebo	8.74	17.38	19	

Exercise VAS val. diff. Tests 1 and 3	Physio + BEMER	15.44	22.67	18	0.6571
	Physio + placebo	11.26	20.90	19	

Oswestry diff. Tests 1 and 3	Physio + BEMER	5.87	9.91	18	0.9773
	Physio + placebo	4.68	14.74	18	

Fatigue GFI diff. Tests 1 and 3	Physio + BEMER	5.04	9.84	13	0.5316
	Physio + placebo	2.80	7.96	12	

Physical functioning nbs diff. Tests 1 and 3	Physio + BEMER	−1.18	5.66	13	0.4034
	Physio + placebo	−1.03	4.11	13	

Role physical nbs diff. Tests 1 and 3	Physio + BEMER	−4.49	11.55	14	0.4105
	Physio + placebo	0.64	10.25	14	

Bodily pain nbs diff. Tests 1 and 3	Physio + BEMER	−6.45	6.28	15	0.1099
	Physio + placebo	−2.44	7.93	18	

General heath nbs diff. Tests 1 and 3	Physio + BEMER	−3.57	4.24	12	0.9441
	Physio + placebo	−2.17	5.57	14	

Vitality nbs diff. Tests 1 and 3	Physio + BEMER	−5.35	6.54	10	0.7085
	Physio + placebo	0.25	6.64	12	

Social functioning nbs. diff. Tests 1 and 3	Physio + BEMER	−1.54	10.11	13	0.6081
	Physio + placebo	−0.56	10.30	18	

Role emotional nbs diff. Tests 1 and 3	Physio + BEMER	−5.36	19.31	13	0.2712
	Physio + placebo	−1.86	14.76	15	

Mental health nbs diff. Tests 1 and 3	Physio + BEMER	−4.36	7.28	9	0.9854
	Physio + placebo	−3.84	7.84	15	

Physical component summary diff. Tests 1 and 3	Physio + BEMER	−2.99	5.57	6	0.9299
	Physio + placebo	−2.06	5.05	10	

Mental component summary diff. Tests 1 and 3	Physio + BEMER	−9.97	2.68	6	0.4874
	Physio + placebo	−1.31	10.13	10	

**Table 3:** Comparison of values before and after treatment of knee osteoarthritis.

Dependent variable	Therapy	Descriptive statistics			*p* values of ANCOVA
		Mean	Std. deviation	*N*	
Resting VAS diff. Tests 1 and 2	Physio + BEMER	15.94	15.67	18	0.9901
	Physio + placebo	20.58	26.68	24	

Exercise VAS val. diff. Tests 1 and 2	Physio + BEMER	18.22	17.17	18	0.3630
	Physio + placebo	25.79	21.55	24	

Fatigue GFI diff. Tests 1 and 2	Physio + BEMER	6.03	6.08	10	0.4270
	Physio + placebo	4.96	9.57	7	

Physical functioning nbs diff. Tests 1 and 2	Physio + BEMER	−2.76	5.25	9	0.6392
	Physio + placebo	−3.83	6.20	17	

Role physical nbs diff. Tests 1 and 2	Physio + BEMER	−13.47	13.53	14	0.7978
	Physio + placebo	−9.48	15.85	18	

Bodily pain nbs diff. Tests 1 and 2	Physio + BEMER	−6.97	6.35	17	0.7382
	Physio + placebo	−6.59	6.04	17	

General heath nbs diff. Tests 1 and 2	Physio + BEMER	−2.96	4.19	13	0.2222
	Physio + placebo	−0.95	3.36	13	

Vitality nbs diff. Tests 1 and 2	Physio + BEMER	−7.64	11.28	14	0.0788
	Physio + placebo	−2.38	9.81	20	

Social functioning nbs diff. Tests 1 and 2	Physio + BEMER	−8.15	10.50	16	0.2459
	Physio + placebo	−5.90	11.92	17	

Role emotional nbs diff. Tests 1 and 2	Physio + BEMER	−13.93	19.70	15	0.8289
	Physio + placebo	−7.96	19.48	14	

Mental health nbs diff. Tests 1 and 2	Physio + BEMER	−5.45	11.67	12	0.9227
	Physio + placebo	−3.72	8.51	19	

Physical component summary diff. Tests 1 and 2	Physio + BEMER	−2.65	4.30	6	0.8054
	Physio + placebo	−5.86	7.68	7	

Mental component summary diff. Tests 1 and 2	Physio + BEMER	−11.58	18.21	6	0.4531
	Physio + placebo	−4.96	7.78	7	

WOMAC “A” diff. 1-2	Physio + BEMER	20.11	19.26	17	0.9221
	Physio + placebo	17.81	17.51	24	

WOMAC “B” diff. 1-2	Physio + BEMER	15.78	18.41	16	0.7126
	Physio + placebo	14.21	20.91	24	

WOMAC “C” diff. 1-2	Physio + BEMER	10.87	15.48	17	0.9848
	Physio + placebo	15.16	14.94	24	

WOMAC total diff. 1-2	Physio + BEMER	13.46	15.10	17	0.8817
	Physio + placebo	15.00	13.83	24	

**Table 4:** Comparison of values before and 3 months after treatment of knee osteoarthritis.

Dependent variable	Therapy	Descriptive statistics			*p* values of ANCOVA
		Mean	Std. deviation	*N*	
Resting VAS diff. Tests 1 and 3	Physio + BEMER	13.03	25.03	20	0.6565
	Physio + placebo	12.2	24.99	20	

Exercise VAS val. diff. Tests 1 and 3	Physio + BEMER	14.85	19.81	20	0.6760
	Physio + placebo	13.30	24.59	20	

Fatigue GFI diff. Tests 1 and 3	Physio + BEMER	4.22	8.54	16	0.0235
	Physio + placebo	−3.30	8.52	11	

Physical functioning nbs diff. Tests 1 and 3	Physio + BEMER	−2.27	6.49	16	0.8051
	Physio + placebo	−3.68	6.96	13	

Role physical nbs diff. Tests 1 and 3	Physio + BEMER	−1.89	13.91	19	0.8724
	Physio + placebo	−5.28	13.50	17	

Bodily pain nbs diff. Tests 1 and 3	Physio + BEMER	−6.41	8.90	18	0.3015
	Physio + placebo	−3.38	6.08	18	

General heath nbs diff. Tests 1 and 3	Physio + BEMER	−5.12	8.08	18	0.4666
	Physio + placebo	−1.43	3.69	12	

Vitality nbs diff. Tests 1 and 3	Physio + BEMER	−5.78	8.61	18	0.0079
	Physio + placebo	2.55	6.98	14	

Social functioning nbs. diff. Tests 1 and 3	Physio + BEMER	−1.47	10.45	17	0.5787
	Physio + placebo	−0.30	11.14	17	

Role emotional nbs diff. Tests 1 and 3	Physio + BEMER	−6.55	13.14	17	0.0371
	Physio + placebo	−1.74	15.98	16	

Mental health nbs diff. Tests 1 and 3	Physio + BEMER	−3.76	12.16	16	0.1842
	Physio + placebo	5.67	8.63	12	

Physical component summary diff. Tests 1 and 3	Physio + BEMER	−4.50	12.57	12	0.6942
	Physio + placebo	−3.20	4.22	5	

Mental component summary diff. Tests 1 and 3	Physio + BEMER	−6.41	11.74	12	0.1940
	Physio + placebo	2.47	11.81	5	

WOMAC “A” diff. 1-3	Physio + BEMER	11.55	18.26	20	0.7254
	Physio + placebo	12.53	21.92	18	

WOMAC “B” diff. 1-3	Physio + BEMER	9.60	21.21	20	0.3888
	Physio + placebo	13.44	31.24	18	

WOMAC “C” diff. 1-3	Physio + BEMER	8.98	20.77	20	0.7020
	Physio + placebo	8.51	21.09	18	

WOMAC total diff. 1-3	Physio + BEMER	8.45	17.92	20	0.4711
	Physio + placebo	10.15	18.97	18	

## Abbreviations

PEMF: Pulsed electromagnetic field
BEMER: Physical vascular therapy
WOMAC: The Western Ontario and McMaster Universities Arthritis Index
VAS: Visual analogue scale
ACR: American College of Rheumatology.
